# Longitudinal growth and health outcomes in nutritionally at‐risk children who received long‐term nutritional intervention

**DOI:** 10.1111/jhn.12306

**Published:** 2015-03-25

**Authors:** D. T. T. Huynh, E. Estorninos, R. Z. Capeding, J. S. Oliver, Y. L. Low, F. J. Rosales

**Affiliations:** ^1^ Abbott Nutrition Research & Development Asia‐Pacific Centre Abbott Laboratories Singapore Singapore; ^2^ Asian Hospital and Medical Centre Manila Philippines; ^3^ Scientific and Medical Affairs Abbott Nutrition Abbott Laboratories Columbus OH USA

**Keywords:** longitudinal growth, long‐term supplementation, oral nutritional supplement, undernutrition

## Abstract

**Background:**

The benefits of short‐term oral nutritional supplementation (ONS) in undernourished children are well‐established. The benefits of long‐term ONS in promoting longitudinal growth and health in children who are at risk of undernutrition have not been reported previously.

**Methods:**

In this 48‐week prospective, single‐arm, multicentre trial, 200 Filipino children aged 3–4 years with weight‐for‐height percentiles from 5th to 25th (WHO Child Growth Standards) were enrolled. Parents received dietary counselling at baseline, and at weeks 4 and 8. Two servings of ONS (450 mL) were consumed daily, providing 450 kcal, 13.5 g protein and micronutrients. Weight, height, dietary intake using 24‐h dietary recalls, and physical activity and appetite using the visual analogue scales were assessed at baseline and weeks 4, 8, 16, 24, 32, 40 and 48. The number of sick days for acute illnesses was collected over the study period.

**Results:**

At baseline, mean age was 41.2 months with 50% being male. Weight‐for‐height percentiles showed the greatest increase in the first 4 weeks (12.1 and 12.8 percentiles, respectively, *P* < 0.0001) and remained significantly higher than baseline (*P* < 0.0001) but were relatively stable from week 4 onwards. Height‐for‐age percentiles increased steadily over time and became significantly higher than baseline from week 24 onwards (*P* < 0.0001). Appetite and physical activity scores at all post‐baseline visits improved from baseline (*P* < 0.0001), and a reduction in the number of sick days from week 16 onwards was also observed (*P* < 0.0001). Higher parental education level, being male and higher baseline weight‐for‐height percentiles were significantly associated with higher ponderal and linear growth over time in repeated measures analysis of covariance.

**Conclusions:**

Intervention consisting of initial dietary counselling and continued ONS helped sustain normal growth after a catch‐up growth in nutritionally at‐risk children.

## Introduction

Poor growth among Filipino children remains a concern despite a decrease in undernutrition among children over the past two decades 1. The causes of undernutrition in this child population are seen to be similar to those reported in other developing countries, including inappropriate feeding practices leading to an inadequate diet, low socioeconomic status and an unhealthy household environment 2, 3.

Poor nutrition in early childhood has been associated with reduced human capital, such as shorter adult height, less schooling and reduced economic productivity, as well as increased risks of chronic diseases 4. In addition, an analysis of Filipino children aged 0–5 years using nationally representative data showed that the total population‐attributable risk for child deaths as a result of undernutrition was 46%, of which 93% was related to mild‐to‐moderate undernutrition 5. Recently, a pooled data analysis involving 53 809 children aged 1 week to 59 months of age from 10 prospective studies in Africa, Asia and South America, including the data from the Philippines, has also demonstrated that even mild anthropometric deficits (−2 ≤ *Z*‐scores < −1 or 2.3rd ≤ percentile < 15th for various growth parameters including weight‐for‐age, height‐for‐age and weight‐for‐height) were associated with a significantly higher risk of mortality in childhood, especially from infectious diseases 6. These findings stressed the importance of identifying effective intervention models to remedy the health consequences and economic impacts of childhood undernutrition.

The treatment of undernutrition in children aims to improve energy and nutrient intake for promoting catch‐up growth 7. Dietary counselling using family foods is considered an integral part of treating undernutrition in children 8. However, the effects of dietary counselling on children's health and nutrition outcomes were shown to vary from little to significant success 9, 10. There are a number of limiting factors that may impede the success of nutrition counselling, such as the intensity of the intervention, the complexity of the behaviours to be changed, timing constraints and the existence of other external constraints such as low food availability 10. By contrast, oral nutritional supplementation (ONS) of macro‐ and micronutrients plus dietary counselling has been shown to be a more effective method for treating and preventing undernutrition in young children compared to dietary counselling alone. In a group of pre‐school‐aged children with evidence of growth faltering (defined as weight‐for‐height percentile below the 25th percentile) and picky eating behaviour, ONS plus dietary counselling over a 3‐month period led to significantly greater improvement in different growth indicators including weight‐for‐age (WAP), height‐for‐age (HAP) and weight‐for‐height percentiles (WHP) compared to dietary counselling alone 11. However, although the short‐term benefits of oral nutritional supplementation in promoting catch‐up growth in undernourished children are well‐established, the effects of long‐term nutritional supplementation are less certain, especially in children who are only mildly undernourished or at the lower end of the normal growth range. Whether the long‐term use of oral nutritional supplements in such children may result in excessive weight gain is also unknown.

The present study aimed to assess the effects of the intervention model consisting of initial dietary counselling and long‐term oral nutritional supplementation of macro‐ and micronutrients over 48 weeks on ponderal and linear growth patterns and related health aspects in 36–48‐month‐old children at risk of undernutrition. Because a preventive approach of treating children at risk of undernutrition has been suggested to be more effective in combating undernutrition in children younger than 5 years of age in populations with a high burden of child undernutrition 12, we chose to investigate a preventive approach of intervening in nutritionally at‐risk children including both mildly undernourished children (WHP percentile between 5 and <15) and those at the lower end of the normal growth range (WHP percentile between 15 and 25).

## Materials and methods

### Study design and participants

The present study was a prospective, multicentre, single‐arm study that was conducted in the city of Manila, Philippines between October 2011 and October 2012. Children were recruited from children health clinics from the Asian Hospital and Medical Centre and the Medical City. Clinically healthy children were eligible for inclusion if they were 36–48 months of age and at risk of undernutrition defined as WHP from the 5th to the 25th based on the WHO Growth Standards. Excluded from the study were children who had a history of preterm delivery, birth weight <2500 g or >4000 g, or current or chronic infections (except for intestinal parasites infection), diarrhoea, acute and chronic hepatitis B or C, HIV or tuberculosis, or a diagnosis of neoplastic diseases, renal, hepatic and cardiovascular diseases, or a diagnosis of congenital or genetic disorder or infantile anorexia nervosa. Because parental obesity is an important risk factor that contributes both genetic and family environmental influences for being overweight in childhood 13, children having an obese parent defined as measured or self‐reported body mass index ≥ 27.5 kg m^–2^ were also excluded.

The study was approved by the Institutional Review Board and the Food and Drug Administration of the Philippines. Written informed consent was obtained from each child's parents or legal guardian. The study was performed in accordance with the ethical principles that had their origin in the Declaration of Helsinki (clinicaltrials.gov number NCT01658267).

### Intervention

Eligible children received three sessions of dietary counselling administered at baseline and weeks 4 and 8 post‐baseline plus two servings of ONS per day for 48 weeks. The dietary counselling was conducted by the study physicians who were trained on a standardised method. The Recommended Energy and Nutrient Intakes for Filipino children aged 4–6 years 14 and materials on good feeding practice from WHO were used as guidelines for preparing the content of the dietary counselling. During the dietary session, parents were advised on food group selection, for which they were provided a list of foods with good protein quality from animal and vegetable sources, foods containing vegetable fat and locally available fatty fishes, staple foods, and also foods with dietary fibres such as beans, lentils and peas. In addition, some portion sizes of cooked foods from different food groups were given to aid parents or caregivers in estimating the adequate amounts to be consumed by the child. Based on WHO guidelines, techniques to enhance the child's eating behaviour with respect to mealtime and feeding environment, meal frequency, limiting consumption of sweetened foods and beverages were also given during the session 15.

Children from 3–4 years of age should be given family foods at three meals each day and twice daily nutritious snacks such as a nutritional supplement, banana or bread 15. The ONS was to be consumed at snack time at mid‐morning and mid‐afternoon or before bedtime. The ONS, commercially available (PediaSure; Abbott Laboratories, Taguig City, Philippines), was used as a tool for improving the overall diet quality. When given twice daily, the ONS provided 450 kcal, 13.5 g of high quality protein, 17.7 g of easily‐digested fat and 59.4 g of carbohydrate and 28 minerals and vitamins (450 mL in total) to meet approximately 30% and 50% of the energy and micronutrient requirements, respectively (Table 1). It also contained *Lactobacillus acidophilus, Bifidobacterium* spp. and fructo‐oligosaccharides.

**Table 1:** Nutrient composition of nutritional supplement and percentage of recommended intakes for Filipino children aged 4–6 years

Nutrients	Nutritional supplement (450 mL)	Percentage of recommended intakes for Filipino children aged 4–6 years (Barba & Cabrera 14) (%)
Energy, kcal	450	32
Protein (g)	13.5	48
Vitamin A (μg)	270	35.5
Vitamin C (mg)	45	150
Vitamin B_1_ (mg)	1.4	230
Vitamin B_2_ (mg)	0.9	150
Niacin (mg)	6.8	97.1
Vitamin B_6_ (mg)	1.2	200
Vitamin B_12_ (μg)	1.4	120
Vitamin D (μg)	9.0	180
Vitamin E (mg)	7.2	144
Folate (μg)	112.5	56
Calcium (mg)	432	78.5
Iron (mg)	6.3	70
Iodine (μg)	43.7	49
Magnesium (mg)	89.1	117
Phosphorus (mg)	373.5	75
Zinc (mg)	3.0	56
Selenium (μg)	14.4	65
Manganese (mg)	0.7	47
Lactobacillus acidophilus (cfu)	3.9 × 10^7^	
*Bifidobacterium* spp. (cfu)	2.45 × 10^6^	
Fructo‐oligosaccharides (g)	1.98	

### Outcome assessments

The compliance of long‐term ONS was assessed and its impact on nutritional status was measured by the changes in weight‐for‐age, weight‐for‐height and height‐for‐age over the study period relative to the degree of compliance with the ONS. Other outcomes include the number of sick days over the study period, parental assessment of child's appetite and physical activity level.

### Compliance assessment

Compliance with the study product was assessed from the product intake records for which parents or caregivers were asked to complete on a daily basis. The compliance was categorised into high, medium and low using the percentage of actual study product intake over the study period with the cut‐offs: 85–115%, 65% to <85% and <65% respectively.

### Anthropometric assessment

Anthropometric measurements were performed by study staff who trained on standardised methods of conducting the measurements. Weight was measured with light clothes and shoes and jackets removed, using electronic weighing scales (Tanita HD380; Tanita, Manila, Philippines) and recorded to the nearest 0.1 kg. Standing height was measured without shoes or hat, using a height measuring gauge (Seca 217; Seca, Hamburg, Germany) and recorded to the nearest 0.1 cm. Weight and height were measured at baseline and all post‐baseline visits at weeks 4, 8, 16, 24, 32, 40 and 48.

### Assessment of growth and nutritional status

Weight‐for‐age, weight‐for‐height and height‐for‐age were expressed as sex‐age‐specific percentiles and *Z*‐scores using the WHO Child Growth Standards. Wasting and being overweight were defined as weight‐for‐height percentiles <2.3rd and >97.7th respectively. In addition, being at risk of wasting and being overweight was assessed as weight‐for‐height between 2.3rd and <25th and between the 85th and 97.7th percentiles, respectively. The rate of weight‐for‐height *Z*‐score gain from baseline to each post‐baseline time point was also calculated.

### Dietary assessment

Dietary intake was collected using 24‐h food recall method by the trained nutritionists from the Food and Nutrition Research Institute in the Philippines (FNRI). At baseline, one 24‐h food recall was completed. Two 24‐h food recalls for two nonconsecutive days were performed at all post‐baseline visits. The first recall was conducted in the weekend prior to the scheduled visit via a telephone interview and the second recall was completed at the visit. After conducting the quality control checks for data accuracy and completeness, dietary intake data were analysed using the Individual Dietary Evaluation Software (ides) developed by the FNRI. For assessing an increase in energy intake from baseline to post‐baseline visits, the mean energy intake was calculated from two recalls for all post‐baseline visits.

### Physical activity and appetite

Parents were asked to rate their child's physical activity and appetite over the last 24 h using the Visual Analogue Scales, scoring 1–10 at baseline and all post‐baseline visits.

### Sick days

From the adverse event (AE) and serious adverse event (SAE) reports collected over the study period as part of normal clinical trial surveillance, we extracted information on the incidences and the number of sick days of common acute illnesses that are associated with childhood morbidities and mortalities, including diarrhoeal and upper and lower respiratory tract infections 6. The total number of sick days for the subject was calculated from the date of onset to the date resolved for each event, as recorded in the AE page. If more than one event occurred on the same date, the date was only counted once in the determination of number of sick days. In the analysis examining the trend in number of sick days over 48 weeks, the number of sick days at both weeks 4 and 8 was combined for equally spaced data points at each 8‐week interval.

### Statistical analysis

All statistical analyses were performed on an intent‐to‐treat basis, using sas, version 9.1.3 (SAS Institute Inc., Cary, NC, USA). Descriptive results, including anthropometric measurements, energy intake, number of sick days, physical activity and appetite scores, were summarised by the mean, median and SD. Categorical variables were summarised by number of subjects (*n*) and as a percentage (%). All continuous variables were checked for normality using the Shapiro–Wilk test. If the Shapiro–Wilk test was significant (*P* < 0.05), the variable was declared to be non‐normal. The nonparametric tests, including the Mann–Whitney *U*‐test and the signed rank test with stepdown Bonferroni adjustment, were used to examine the differences between two groups for continuous outcome variables with non‐normal distribution.

The association between repeated measures of WHP and HAP over the study period with sociodemographic factors, including age (months), sex and parental education level, baseline nutritional status such as mild wasting (WHP < 15) versus normal (WHP ≥ 15), study site, study visits and the compliance with ONS consumption, expressed as a percentage of ONS consumption, was assessed using repeated measures analysis of covariance (ancova). In addition, ancova was used to assess whether these factors have an impact on the number of sick days or parent‐reported appetite and physical activity scores over the study period.

## Results

### Baseline characteristics of subjects

Two hundred and twenty‐four subjects were screened, of whom 200 were enrolled. Of the 200 enrolled subjects, one subject did not meet the eligibility criteria for age group and never consumed the study product. This results in the intent‐to‐treat population of 199 subjects.

Baseline characteristics of children and their parents are shown in Table 2. The mean age was 41.2 months and 50% were male. In general, children had ponderal growth faltering rather than linear growth faltering problem because the mean weight‐for‐age percentile was the lowest among growth indicators. Males were heavier than females at baseline (*P* < 0.0001), whereas mean height was comparable between the two sexes. In addition, males had significantly greater mean percentiles of weight‐for‐height at baseline than females. In terms of parental characteristics, there were more than 90% of children whose mothers attended high school and/or college or university.

**Table 2:** Baseline characteristics of children participated in the present study

Baseline characteristics^a^	Study group (*n* = 199)	*P*‐value
Age (months)	41.2 (3.59)	
Sex		
Male	99 (49.7)	
Maternal age (years)	30.0 (6.19)	
Maternal education level		
College/University or Higher	84 (42.2)	
High School	114 (57.3)	
Secondary School	0	
Primary School	1 (0.5)	
Weight (kg)	12.4 (0.98)	
Male	12.7 (0.90)	<0.0001^b^
Female	12.2 (0.98)	
Height (cm)	93.3 (3.94)	
Male	93.7 (4.06)	0.1049^b^
Female	92.8 (3.79)	
Weight‐for‐height percentiles	15.9 (7.29)	
Male	17.0 (7.64)	0.0228^c^
Female	14.8 (6.79)	
Weight‐for‐age percentiles	8.9 (8.54)	
Male	9.8 (8.60)	0.0712^c^
Female	8.1 (8.46)	
Height‐for‐age percentiles	14.2 (17.0)	
Male	15.3 (18.5)	0.7083^c^
Female	13.1 (15.4)	

a Data are presented as the means (SD), except for sex, which is presented as a percentage.

b *P*‐value is from a *t*‐test.

c *P*‐value is from the Mann–Whitney *U*‐test.

### Compliance

**Figure 1:**
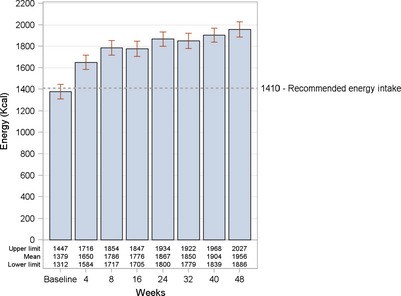
Mean energy intake at baseline and each post‐baseline visit. *P* < 0.0001 for each post‐baseline visit compared to baseline. The *P*‐value is from a signed rank test when controlling for the total number of seven comparisons using stepdown Bonferroni adjustment.

There was high compliance because 100% of children were reported to consume at the recommended dose of ONS with at least 85% of two servings on a daily basis. In accordance with a high compliance with ONS consumption, there were improving trends in total energy intake over the study period (*P* < 0.0001) (Fig. 1).

### Changes in growth indicators over time

**Figure 2:**
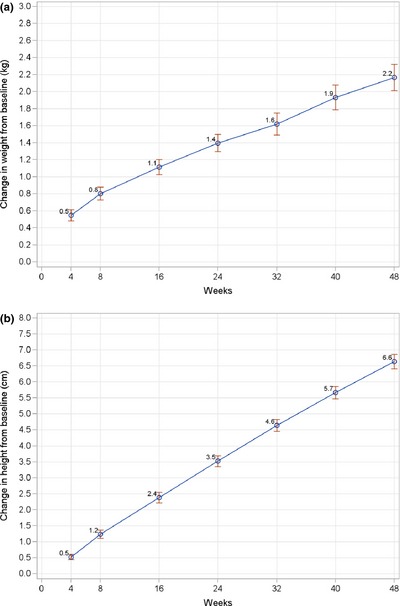
Line graphs of mean change in weight (kg) (a) and height (cm) (b) from baseline to each post‐baseline visit. Data points represent mean values; error bars represent 95% confidence intervals of the mean. *P* < 0.0001 for all post‐baseline visits compared to baseline; *P*‐value is from a paired *t*‐test.

Because of the lack of diversity in compliance categories, data were analysed and presented as a single group. In terms of weight change, children had more weight gain for the first 8 weeks of the study (0.5 and 0.3 kg on average in the first and second 4 weeks, respectively) than for the remaining period (0.2–0.3 kg per 8 weeks) (Fig. 2a). By contrast, height steadily increased over the study period, with a mean rate of 0.5 cm every 4 weeks (Fig. 2b).

**Figure 3:**
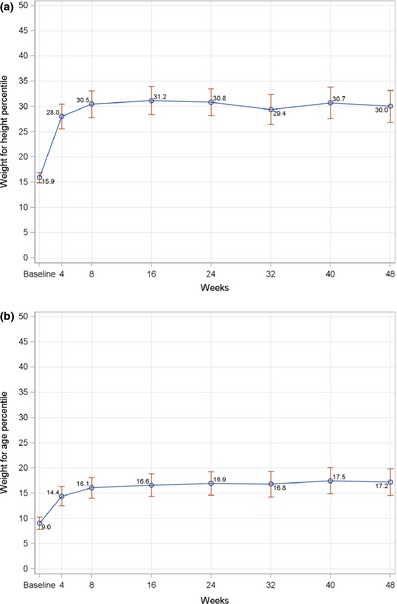
Line graphs of mean change in weight‐for‐height (a) and weight‐for‐age (b) percentiles from baseline to each post‐baseline visit. Data points represent mean values; error bars represent 95% confidence intervals of the mean. *P* < 0.0001 for all post‐baseline visits compared to baseline; *P*‐value is from a signed rank test using stepdown Bonferroni adjustment controlling for all pairwise comparisons for changes from baseline.

**Figure 4:**
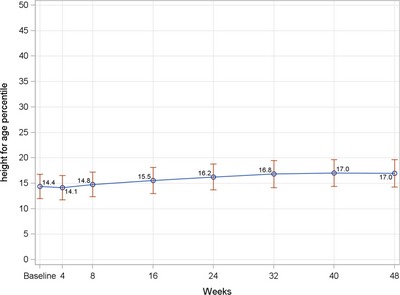
Line graphs of mean change in height‐for‐age from baseline to each post‐baseline visit. Data points represent mean values; error bars represent 95% confidence intervals of the mean. *P* < 0.05 from week 16 onwards compared to baseline; *P*‐value is from a signed rank test using stepdown Bonferroni adjustment controlling for all pairwise comparisons for changes from baseline.

**Figure 5:**
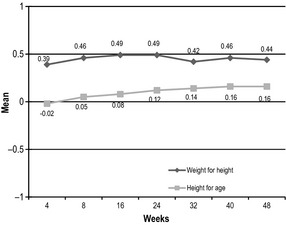
Mean changes in weight‐for‐height and height‐for‐age *Z*‐scores from baseline to each post‐baseline time point.

Various anthropometric indices were used to assess longitudinal growth that includes both ponderal and linear growth. The patterns observed for these growth indices were similar and characterised by two phases, including catch‐up growth and maintenance of growth. Children had the greatest increases in mean WHP and WAP in the first 4 weeks of the study compared to the changes between consecutive visits. At least 70% of children had an increase in WHP and WAP during this period (data not shown). The rates of percentiles gain slowed down after week 4 and were relatively stable for the rest of the study (Fig. 3). Percentiles at all post‐baseline visits were significantly higher than baseline (*P* < 0.0001). In terms of linear growth, children steadily increased HAP over time, except for the period of week 40 to week 48, and this reached statistical significance from week 16 onwards compared to baseline (Fig. 4). At week 48, using WHO definitions for assessing undernutrition and overnutrition, four (2.1%) children and one child (0.5%) were categorised as wasting and being overweight, respectively (data not shown). Although these wasting children were good compliers with ONS consumption (85–100%) and had an improved energy intake at most of post‐baseline visits, a further decrease in WHP was possibly the result of acute illnesses in two children (total number of sick days of 8–10 day over the study period), whereas there was a greater increase in HAP and a further decrease in WAP compared to baseline in the other children leading to smaller WHP. In terms of growth velocity, the mean increase in WH *Z*‐scores at all post‐baseline time points was below 0.67, which is the recommended cut‐off point for defining rapid weight gain (Fig. 5) 16.

**Figure 6:**
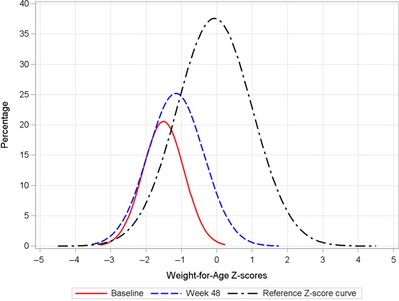
Weight‐for‐age *Z*‐scores distribution curves at baseline and week 48.

**Figure 7:**
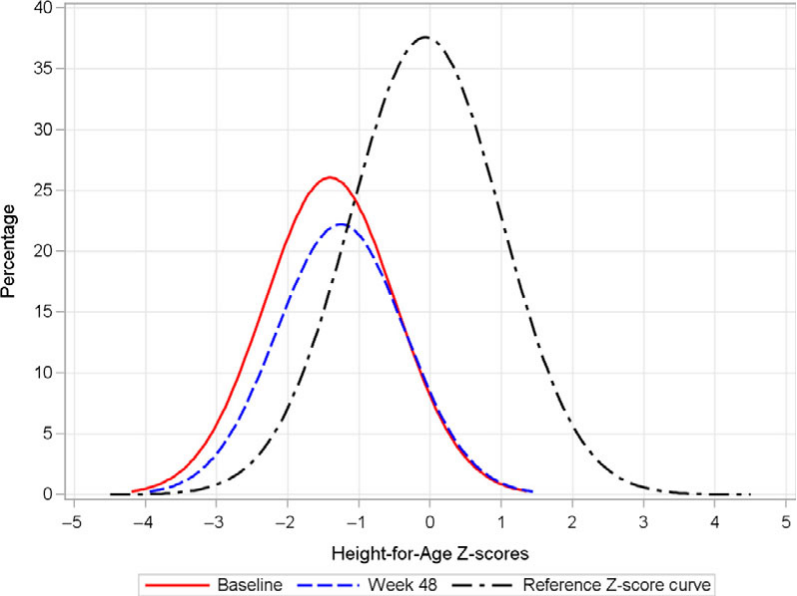
Height‐for‐age *Z*‐scores distribution curves at baseline and week 48.

Figures 6 and 7 show the weight‐for‐age and height‐for‐age *Z*‐score distributions at baseline and week 48. Both *Z*‐score distributions at week 48 were shifted to the right and towards the normal distribution, representing the distribution of the reference population of WHO Child Growth Standards 17. There was a further shift toward the central value (0 *Z*‐score) or greater improvement in *Z*‐score for weight‐for‐age than for height‐for‐age. A similar *Z*‐score distribution was seen for weight‐for‐height (data not shown).

### Sick days, physical activity and appetite scores

**Figure 8:**
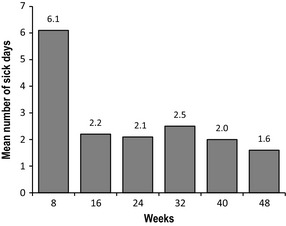
Mean number of sick days at an 8‐week interval over 48‐week period. *P* < 0.0001 for each post‐week 8 visit; *P*‐value is from a signed rank test using stepdown Bonferroni adjustment controlling for all pairwise comparisons versus week 8.

**Figure 9:**
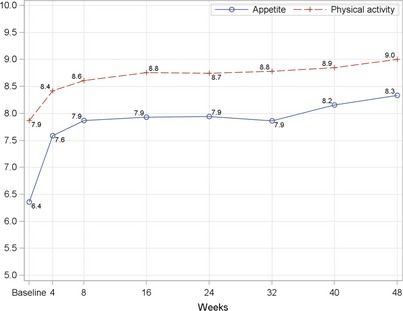
Physical activity and appetite scores at baseline and each follow‐up visit. *P* < 0.0001 for all post‐baseline visits when compared with baseline; *P*‐value is from a signed rank test using stepdown Bonferroni adjustment controlling for all pairwise comparisons versus baseline.

The trend in number of sick days over the 48‐week period was examined. When the first 8 weeks of intervention were used as a reference, the number of sick days of each post‐week 8 visit was significantly reduced compared to the reference (*P* < 0.0001) (Fig. 8). In addition, parents reported an improvement in appetite and physical activity levels from baseline to each post‐baseline visit (*P* < 0.0001 for each post‐baseline visit) (Fig. 9).

### Factors associated with ponderal and linear growth over time

Several factors were investigated to determine their association with ponderal and linear growth. Several factors were shown to have significant positive associations with an increase in WHP over the study period, including lower WHP in the normal range at baseline (*P* < 0.0001), being male (*P* = 0.0006), highest parental education of college or university or higher (*P* = 0.0002) and better compliance with ONS consumption (*P* = 0.0021) (Table 3). Similarly, these are also significant positive predictors for the development of HAP over time, except for the overall ONS compliance (Table 4). By contrast, there are no associations between the examined factors and the reduced number of sick days or improved appetite or physical activity scores (data not shown).

**Table 3:** Relationship between weight for height percentile and sociodemographic factors over time

Effect	Least squares mean	Standard error	*P*‐value
Baseline nutritional status			
Mildly wasting (WHP < 15)	19.75	0.91	<0.0001
Normal (WHP ≥ 15)	35.83	0.85	
Weeks			
4	25.68	1.40	0.5812
8	28.13	1.40	
16	29.06	1.40	
24	28.54	1.40	
32	26.89	1.40	
40	28.43	1.40	
48	27.78	1.40	
Sex			
Female	26.07	0.88	0.0006
Male	29.51	0.87	
Baseline age (months)			0.1670
Study site			
Asian Hospital and Medical Center	28.93	0.55	0.1221
The Medical City	26.65	1.35	
Highest parental education			
High school	25.91	0.88	0.0002
College/University or Higher	29.66	0.88	
Compliance with ONS consumption			
Overall percentage of ONS consumed			0.0021

*P*‐value is from repeated measures ancova. ons, oral nutritional supplementation; whp, weight‐for‐height percentile.

**Table 4:** Relationship between height for age and sociodemographic factors over time

Effect	Least squares mean	Standard error	*P*‐value
Baseline nutritional status			
Mildly wasting (WHP < 15)	4.65	0.54	<0.0001
Normal (WHP ≥ 15)	32.08	0.68	
Weeks			
4	16.61	0.95	0.1473
8	17.23	0.95	
16	18.03	0.95	
24	18.75	0.95	
32	19.19	0.95	
40	19.36	0.95	
48	19.39	0.95	
Sex			
Female	16.95	0.61	<0.0001
Male	19.78	0.60	
Baseline age (months)			0.1187
Study site			
Asian Hospital and Medical Center	20.76	0.38	<0.0001
The Medical City	16.0	0.92	
Highest parental education			
High school	17.28	0.61	0.0015
College/University or Higher	19.45	0.60	
Compliance with ONS consumption			
Overall percentage of ONS consumed			0.9240

*P*‐value is from repeated measures ancova. ons, oral nutritional supplementation; whp, weight‐for‐height percentile.

## Discussion

Conventionally, nutritional intervention targets children with moderate and severe undernutrition rather than those with mild undernutrition because of its significant impact on health and growth, both short and longer term 18, 19. On the other hand, children at the lower end of the normal growth are considered normally nourished and therefore are not the focus for nutritional intervention. The present study shows that, in these nutritionally at‐risk children with low anthropometric values, the intervention model consisting of initial dietary counselling and long‐term use of an oral nutritional supplement promoted ponderal growth in the catch‐up growth phase and linear growth during the maintenance growth phase, thereby promoting and sustaining proportional growth. In addition, we observed health benefits on the number of sick days, appetite, physical activity levels and thus overall health in pre‐school‐aged children in a developing country. These benefits have been achieved with a low risk of excessive weight gain. This confirms the hypothesis that the preventive approach of targeting children at risk of undernutrition using an intervention model consisting of initial dietary counselling and continued ONS is safe and effective with respect to improving nutritional status and health in children under 5 years of age.

This study showed a gain in weight (WAP) during the catch‐up phase, followed by a gain in height (HAP). This growth pattern has been observed in studies of catch‐up growth in severely undernourished children who demonstrated weight gain preceding height gain with the onset of supplementation 20, 21. In the present study, however, none of children were moderately or severely undernourished. Thus, their growth represents a pattern of saltation and stasis as characterised by Lampl 23, indicating a robust adaptive strategy, by which populations adjust growth patterns accordingly to environmental factors such as available diet. In this regard, it demonstrates the adequacy of the diet in providing the energy and nutrients necessary for growth. Golden 7 has noted that a gain in height is a better indicator of the effectiveness of the nutritional intervention than weight gain alone. This is because weight gain is frequently simply a result of a positive energy balance, whereas height gain only occurs when diets contain necessary nutrients to support syntheses of skeletal tissue and accompanying lean tissue 7. According to Golden 7, these nutrients are classified as the growth nutrients comprising the building blocks of tissues and are necessary for almost all biochemical pathways in which a lack of any of these nutrients will stop the child from growing. Among the available nutrition intervention methods, dietary counselling using family foods for treating undernutrition in children is considered the first line of treatment, although its shortcomings have also been recognised when applied in the setting of developing countries. In addition to the challenges in procuring dietary diversity and in consuming adequate amounts to meet nutrient intake recommendations, plant‐based diets are known to contain relatively high anti‐nutrients, a low nutrient bioavailability for some nutrients such as iron, zinc and vitamin A, and a limited source of active compounds that are available in animal source foods 12. These challenges have contributed to the limited success of relying on dietary counselling using family foods alone to achieve adequate weight and height growth in children at nutritional risk 12, 18.

Growth of children in the present study as measured by WHP demonstrated a healthy gain in body weight because it consists of initial catch‐up growth followed by growth maintenance phase. Although children who were at the lower end of the normal growth range at baseline had greater increases in WHP compared to those with mild wasting, time was not significant in the model of factors associated with WHP over time, especially when the least squares means were comparable from week 8 onwards, suggesting that the increased WHP is stable after week 8. In addition, there was only one child (0.5%) who was categorised as overweight by the end of the study. Because rapid weight gain during childhood was shown to be associated with obesity development in later life, it is recommended that the 0.67 *Z*‐score variation should be used to evaluate rapid growth as a risk factor of later obesity 17. The score variation is based on the difference between the 25th, 50th and 75th percentiles of a growth chart 17. Using this definition, the amount of weight gain in these children within the first 4 weeks of intervention (0.26 *Z*‐scores or 5.5 percentiles and 0.39 *Z*‐scores or 12.2 percentiles, respectively) was considered safe at the same time as helping nutritionally at‐risk children regain growth normality.

In addition to being at the lower end of the normal range, which was associated with the improved growth over time as discussed above, other factors were shown to have an impact on ponderal and linear growth over time, including a higher parental educational level and being male. The findings in the present study were in accordance with the existing literature on the importance of parental education in child's health. Higher parental education levels were shown to be associated with protective caregiving behaviours and a lower risk of child malnutrition in the Philippines, Bangladesh and Indonesia 3, 23. In the present study, males were better nourished than females at baseline and also had significantly greater increase in weight‐for‐height and height‐for‐age percentiles over the study period. This sex difference in growth was previously reported in a longitudinal cohort study from birth to 24 months in Filipino children 24. Although an individual's sex may modulate the responses of growth to nutritional intervention as shown in young infants during the first 6 months of life 25, a male sex preference that appears to be common in Asian countries may have an impact on growth response to the intervention because they may receive better family's resources compared to females.

In the present study, we observed a significant reduction in the number of sick days and a significant increase in the appetite and physical activity scores over the study period. Sociodemographic factors were shown not to be associated with these improved outcomes in the repeated measures ancova analysis. The absence of a control group limited our ability to establish a definitive causal relationship between the intervention and these improved outcomes. Nonetheless, the ONS provided at least 50% of the requirements for nutrients known to be critical for improvement and maintenance of the immune system, such as zinc, vitamin E, vitamin A and selenium, especially when high compliance with ONS consumption was reported in 100% of children 7, 26, 27. This may have preventive implications in providing protection against common infections of respiratory and gastrointestinal tracts in children from developing countries. Compared to those children who live in a clean environment or are not under stress, children who live in crowded or flooded areas such as the children in the present study are advised to consume a diet containing higher protective nutrients 7. In addition, as reported by parents, children in the present study also had a significant increase in appetite and physical activity scores. It is possible that adequate intake of certain micronutrients such as zinc and iron may have a positive impact on appetite because they have been shown to be involved in appetite regulation 28, 29 and improvement of appetite in young children who have evidence of growth faltering 30, 31.

It is noteworthy that the present study was carried out in children aged 3–48 months, which is beyond the ‘first 1000 days window’. It is well‐accepted that nutritional intervention should be implemented during the ‘first 1000 days window’ for better outcomes on child growth and development, whereas less emphasis is placed on nutritional intervention after this window of opportunity because of a modest impact on growth, functionality and health outcomes 32, 33, 34. However, our intervention beyond 1000 days has demonstrated significant benefits on growth, functionality and health outcomes, providing some evidence on the merit of extending the window of intervention beyond 1000 days. Indeed, analyses on longitudinal data on growth from birth cohorts in Brazil, Guatemala, India, the Philippines and South Africa have shown that catch‐up growth may still occur after age of 2 years 35. This is also supported by analysis using the absolute height‐for‐age difference over time, which showed that 70% of height deficit at 60 months was a result of faltering from conception to 24 months, whereas 30% was a result of continued increases in deficit from age 2–5 years 33. Thus, our results add to the body of evidence supporting the proposal that appropriate nutritional intervention in children beyond 2 years old could still be effective and important for minimising the negative impact of growth retardation on both short‐ and long‐term health consequences 7.

The present study had some limitations. First, this is a single‐arm clinical trial with no control group. We expected that there would have been a relative distribution in compliance among the enrolled subjects from low, medium to high compliance. However, the compliance with ONS was 100% in this population, which did not allow for a comparison of growth across compliance levels. We acknowledge that this is a major study limitation and the inclusion of an active control group should be more advantageous for illustrating the causal effects of the intervention on the benefits observed. Nonetheless, given the clear causal link between poor nutrition and faltering growth 36, as well as previously proven benefits between ONS in improving growth among nutritionally at‐risk children 11, 37, the probability that the ONS resulted in the health benefits observed in the present study is high. Additionally, the availability of the well‐established WHO growth standards enables us to compare the growth of children in the present study against the standards and determine the positive effect of the intervention. Second, the high compliance with ONS may be subject to biases because it was measured using parental self‐reporting. However, the high compliance observed in the present study is supported by the increased energy intake through dietary assessment and the increase in both ponderal and linear growth.

In conclusion, the present study shows that nutritional intervention combining initial dietary counselling and long‐term oral nutritional supplementation promoted an initial catch‐up growth in weight and also improved linear growth during the growth maintenance phase, thereby helping to sustain proportional growth in children at risk of undernutrition. Given the positive results of the present study showing that ONS in the form of energy, macro‐ and micronutrients can be a useful tool in the management of child undernutrition, further research is warranted to evaluate the effectiveness of including ONS in the prevention intervention models on promoting growth and health in nutritionally at‐risk children using more robust study designs, such as those including an active control group receiving a placebo supplement. Evaluation of the cost‐effectiveness of such interventions in improving human capital for the society will also provide useful insights into potential public health implications.

**Conflict of interests, source of funding and authorship**

DTTH, JSO, YLL and FJR are employees of Abbott.

Abbott Nutrition provided funding for the study.

DTTH and FJR conceived and designed the study. EE and RZC were responsible for subject recruitment and data collection. DTTH, JSO, YLL, EE, RZC and FJR participated in data analysis and interpretation. DTTH, EE, RZC, JSO, YLL and FJR drafted the manuscript. All authors critically reviewed the manuscript and approved the final version submitted for publication.
